# Early dengue outbreak detection modeling based on dengue incidences in Singapore during 2012 to 2017

**DOI:** 10.1002/sim.8535

**Published:** 2020-03-30

**Authors:** Piao Chen, Xiuju Fu, Stefan Ma, Hai‐Yan Xu, Wanbing Zhang, Gaoxi Xiao, Rick Siow Mong Goh, George Xu, Lee Ching Ng

**Affiliations:** ^1^ Delft Institute of Applied Mathematics Delft University of Technology Delft the Netherlands; ^2^ Institute of High Performance Computing Singapore; ^3^ Epidemiology & Disease Control Division Ministry of Health Singapore; ^4^ School of Electrical and Electronic Engineering Nanyang Technological University Singapore; ^5^ Environmental Health Institute National Environment Agency Singapore

**Keywords:** generalized additive model, statistical process control, public health surveillance, EWMA control chart

## Abstract

Dengue has been as an endemic with year‐round presence in Singapore. In the recent years 2013, 2014, and 2016, there were several severe dengue outbreaks, posing serious threat to the public health. To proactively control and mitigate the disease spread, early warnings of dengue outbreaks, at which there are rapid and large‐scale spread of dengue incidences, are extremely helpful. In this study, a two‐step framework is proposed to predict dengue outbreaks and it is evaluated based on the dengue incidences in Singapore during 2012 to 2017. First, a generalized additive model (GAM) is trained based on the weekly dengue incidence data during 2006 to 2011. The proposed GAM is a one‐week‐ahead forecasting model, and it inherently accounts for the possible correlation among the historical incidence data, making the residuals approximately normally distributed. Then, an exponentially weighted moving average (EWMA) control chart is proposed to sequentially monitor the weekly residuals during 2012 to 2017. Our investigation shows that the proposed two‐step framework is able to give persistent signals at the early stage of the outbreaks in 2013, 2014, and 2016, which provides early alerts of outbreaks and wins time for the early interventions and the preparation of necessary public health resources. In addition, extensive simulations show that the proposed method is comparable to other potential outbreak detection methods and it is robust to the underlying data‐generating mechanisms.

## 1 INTRODUCTION

Dengue is an arthropod‐borne viral disease transmitted by the *Aedes aegypti* and *Aedes albopictus* mosquitos. According to a report by the World Health Organization,^1^ dengue transmission occurs in more than 100 countries with 50 million dengue infections worldwide. In Singapore, dengue is endemic and with year‐round presence due to the tropical climate and rapid urbanization, and it presents a serious economic and disease burden. A great deal of resources (eg, manpower and money) have been allocated for dengue every year and the annual cost was more than US$85 million.^2^

Because of the lack of dengue vaccines, current dengue prevention measure in Singapore focuses on vector control, for example, controlling mosquito populations.^3^ Statistical models have shown to be useful tools in aiding the vector control measure. For example, a least absolute shrinkage and selection operator model has been developed in Singapore to forecast the weekly dengue incidence over a 3‐month horizon.^4^ The model has become part of Singapore's dengue control program and shown its usefulness in planning and resource allocation.^5^ In the literature, there are a multitude of other dengue forecasting models. For example, a multiple linear regression model was used to predict dengue incidences in Mexico;^6^ a Poisson regression model was used for the Singapore cases;^7^ a seasonal autoregressive integrated moving average model was developed for dengue prediction in Guadeloupe, French West Indies;^8^ a wavelet time series model was proposed to predict dengue incidences across geographic regions of Peru.^9^

One problem associated with these forecasting models is that they only focus on prediction accuracy over a certain period but cannot detect dengue outbreaks, that is, rapid and consecutive increases of dengue incidences that deviate from the normal endemic pattern.^10^ During dengue outbreaks, there is not only rapid increase of dengue incidences but also large‐scale spread within a short period, which challenges public health resources, especially if it coincidentally occurs with other emerging infectious diseases. Early detection of outbreaks is very important for an effective disease surveillance system, which targets to detect signals of disease outbreaks earlier before it bursts into larger‐scale infections and spread. The early prediction of outbreaks will gain time to facilitate cost‐effective control to substantially reduce the risk of medical complications and fatal cases, and prepare sufficient public health facilities and resources. One common method for outbreak detection is to directly use the forecasting models.^11^ For example, it is possible to apply the aforementioned dengue forecasting models to predict the weekly dengue incidences for the next several weeks and then to check whether there are unusually large values. However, the accuracy of most forecasting models decreases sharply as the time horizon for forecasts increases.^4^ As a result, the forecasting models may overemphasize on the prediction accuracy and only be useful in detecting dengue incidences in the immediate future. In other words, it may not be able to identify the dengue outbreak patterns which indicate the rapid increase of dengue incidences and the large scale of incidences exceeding the normal trends observed in the near term.

In addition, there is a lack of clear definition of abnormal number of incidences, which calls for models to provide quantitative evaluation of the dramatic increase of dengue incidences considering historical patterns, which could guide the health and environmental agency to carry out proactive control plan and allocate sufficient resources for reducing dengue incidences. Here to tackle with the challenging task, we propose to use the statistical control chart, which has been extensively used in detecting product defectives in a manufacturing process.^10^, ^12^ The disease surveillance data, however, are much more complex than the industrial data. In a typical disease surveillance dataset, the conventional assumptions for the use of a control chart, for example, normality, independence and stationary, are often violated.^10^ To deal with these problems, a two‐step framework for sequential detection of disease outbreak is proposed in this study. Throughout this study, weekly counts of dengue cases in Singapore from 2006 to 2017 are used as a case study. The detailed information of the dataset will be introduced in Section 2.

In the first step, we develop a generalized additive model (GAM) based on the weekly count data from 2006 to 2011. The GAM is widely recognized as a flexible prediction model and its applications can be found in a variety of areas such as ecology,^13^ energy,^14^ and bio‐surveillance systems.^15^ The proposed forecasting procedure is novel in the following three aspects. First, data are incorporated to train the GAM only if the weekly peak incidence in that year is below the annual threshold determined by the Ministry of Health (MOH) of Singapore at the beginning of each year. This procedure approximately selects the normal (or in‐control) dengue data so that the trained GAM could be used for outbreak detection. Second, the proposed GAM focuses on one‐week‐ahead forecasting instead of a long‐time horizon forecasting. As such, a higher prediction accuracy of dengue incidences in the normal pattern is expected. Lastly, the use of a GAM usually requires the response variables (eg, weekly dengue count) being independent so that the errors are also independent. However, the weekly dengue time series are highly correlated and a naive application of the GAM may lead to problematic estimates. To overcome this difficulty, the autoregressive and moving average terms are introduced to the proposed GAM to achieve a reliable estimation.

Once the tailored GAM was developed, it serves as the one‐week‐ahead forecasting model in the second step. The predicted weekly counts are then sequentially added into the monitoring algorithms. In this study, we use the exponentially weighted moving average (EWMA) control chart as the monitoring algorithm, and an alarm of outbreak is triggered once the monitoring statistic exceeds the control limit, which is computed based on the design of the control chart. Because the EWMA procedure consists of a weighted average of all observed data available at the current time point, it is able to detect moderate and persistent shift of the monitoring process.^12^ For example, the EWMA control charts have been extensively used in detecting changes in manufacturing processes.^16^, ^17^ In public health surveillance, the EWMA control charts have also been occasionally used in detecting disease outbreaks.^18^, ^19^ However, most of these applications directly monitor the disease count data,^19^, ^20^ and hence the conventional EWMA control charts which builds on the normally distributed data cannot be used. On the other hand, by taking advantage of the proposed GAM, we use the conventional EWMA chart to monitor the residuals, that is, the (log‐transformed) difference between the predicted weekly incidence and the observed one. This procedure is thus much easier for practitioners to follow and implement.

The remainder of the paper is organized as follows. Section 2 introduces the weekly dengue count data in Singapore during 2006 to 2017. Section 3 proposes a GAM model to deal with the dengue data in 2006 to 2011. In Section 4, an EWMA control chart is proposed to give early signals of dengue outbreaks in 2012 to 2017. In Section 5, simulations are conducted to compare the proposed detection procedures with some commonly used methods in the literature. At last, conclusion is given in Section 7.

## 2 THE DATA

**Figure 1:**
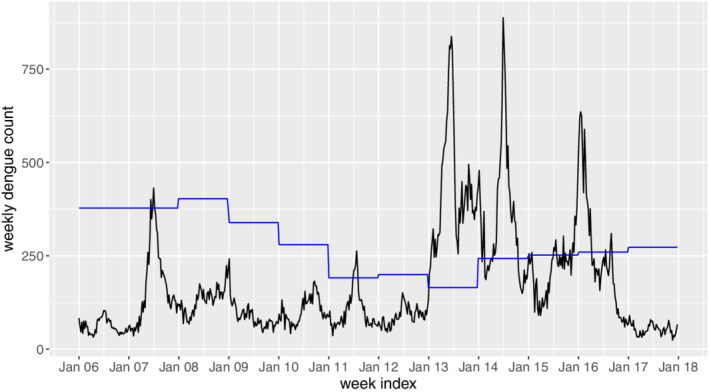
Weekly dengue counts during 2006 to 2017 in Singapore. The blue line denotes the threshold determined by Ministry of Health [Colour figure can be viewed at wileyonlinelibrary.com]

Singapore is a hotbed for the dengue disease due to its tropical climate and urbanized environment. The Singapore government has set up detailed dengue data collecting programs and published the weekly statistics on the official website of MOH.1
https://www.moh.gov.sg/
Figure 1 shows the weekly dengue fever data (eg, counts of notified cases per week) during 2006 to 2017, and Table 1 shows some summary statistics of the weekly dengue data in each year.

**Table 1:** Summary statistics (mean [Mean], SD, threshold by Ministry of Health [Threshold], number of exceeding weeks [Duration] and peak weekly incidence [Peak]) for weekly dengue incidences in 2006 to 2017

Year	2006	2007	2008	2009	2010	2011	2012	2013	2014	2015	2016	2017
Mean	60.2	170.2	132.9	87.0	103.7	102.5	88.5	425.0	345.4	217.2	252.1	53.0
SD	19.5	106.4	34.7	32.5	35.2	51.1	24.7	166.3	174.1	75.8	159.9	14.7
Threshold	378	378	403	339	280	191	200	165	243	252	260	273
Duration	0	3	0	0	0	5	0	51	33	15	17	0
Peak	104	432	224	242	182	263	151	838	888	459	636	90

Evidently, there are several severe outbreaks from 2013 to 2016. According to the MOH annual reports,^3^, ^21^, ^22^, ^23^ 2013 to 2016 have been consistently seen as eventful years for dengue. At peak years, the incidences of dengue may exceed 800 cases within a week, which will challenge the resilience of public health system if there are sudden surge of other diseases as well. Hence, it would be extremely helpful if early warnings of dengue outbreaks in these years can be given. In the literature, an outbreak is often defined as the dengue incidence exceeding a threshold, which can be calculated based on the mean and SDs during past few years.^24^ In Singapore, such a threshold is also adopted by MOH. In specific, the dengue incidence threshold in Singapore is calculated as the sum of the mean weekly count and 2 SD during the past 5 years, with some possible modifications. The blue line in Figure 1 shows the annual dengue thresholds published by MOH, Singapore.

Given the thresholds, an outbreak signal may be generated when there is an exceedance of the weekly dengue incidence. However, as argued by Brady et al,^24^ the capability of the threshold‐based outbreak definition is under doubt. For example, the sporadic exceedances in 2015 and 2016 are treated as outbreaks by the blue line in Figure 1, which may mislead the public health officers. On the other hand, the control chart is able to generate outbreak signals automatically. Due to its appealing statistical properties, the control chart generates outbreak signals when there are rapid and consecutive increases of dengue incidences that deviate from the normal pattern. Therefore, we propose to use the control chart to detect dengue outbreaks in this study. Following the tradition in the context of statistical process control,^12^ we divide the whole dataset into two different phases. In phase I, we consider the data in 2006 to 2011, as they exhibit a relatively stable in‐control pattern. A GAM is then developed based on the phase I data. In phase II, the weekly counts in 2012 to 2017 are considered, and the major goal is to monitor the process online to detect possible anomalies and predict outbreaks based on the historical patterns.

## 3 PHASE I MODELING

In this section, a tailored GAM is proposed for one‐week‐ahead dengue count prediction based on the phase I data. Let *Y* be a response variable (eg, weekly dengue count) and $X={\left(X_{1},\ldots ,X_{m}\right)}^{\prime }\in R^{m}$ be a vector of covariates. The GAM^25^ assumes that

(1) $$g(E[Y|X])=f_{0}+\overset{m}{\underset{j=1}{\sum }}f_{j}(X_{j}),$$

where *g* is an appropriate link function, $f_{0}\in R$ is a constant and *f*_*j*_,*j*=1,…,*m* are smooth functions. Given a sample (*Y*_1_,**X**_1_),⋯,(*Y*_*n*_,**X**_*n*_), the unknown *f*_*j*_'s can be estimated by using the generalized local scoring algorithm.^25^ As a result, E[Y|X] can be estimated as $g^{-1}({\hat{f}}_{0}+\sum_{j}{\hat{f}}_{j}(X_{j}))$. Because the GAM is very flexible in describing the dependence of the response on the covariates, it has been widely used as a prediction model in statistical applications.^13^, ^15^

### 3.1 A modified GAM

A successful application of the GAM in (1) requires the model residuals to be independent, which largely depend on the independence of the response variables *Y*_*t*_'s.^26^ However, as the weekly counts of dengue are observed along time, they are highly correlated. In such cases, a plain application of (1) may lead to an unstable estimation and unreliable inputs for the EWMA charts. To deal with it, we need to incorporate the autoregressive moving average (ARMR) terms to the GAM.

Our idea is motivated by the generalized ARMA models proposed by Benjamin et al,^27^ where the authors incorporate the ARMA terms to a generalized linear model (GLM). Adopting the similar token, we incorporate the ARMA terms to the GAM as follows. Let $\mu_{t}=E[Y_{t}|X_{t},\ldots ,X_{1};Y_{t-1},\ldots ,Y_{1}]$, *t*=1,…,*n* and we introduce an ARMA component τ_*t*_ as

(2) $$\tau_{t}=\overset{p}{\underset{i=1}{\sum }}g_{i}(Y_{t-i},X_{t-i})+\overset{q}{\underset{i=1}{\sum }}s_{i}(Y_{t-i},\mu_{t-i}),$$

where *g*_*i*_'s and *s*_*i*_'s are smooth functions representing the AR terms and the MA terms, respectively. Then, the corresponding GAM becomes

(3) $$\begin{array}{ll} g(\mu_{t}) & =f_{0}+\overset{m}{\underset{j=1}{\sum }}f_{j}(X_{tj})+\tau_{t}\;\\ & =f_{0}+\overset{m}{\underset{j=1}{\sum }}f_{j}(X_{tj})+\overset{p}{\underset{i=1}{\sum }}g_{i}(Y_{t-i},X_{t-i})+\overset{q}{\underset{i=1}{\sum }}s_{i}(Y_{t-i},\mu_{t-i}), \end{array}$$

where *p* and *q* are the orders used in the AR and MA terms, respectively.

The proposed GAM in (3) can be treated as a mixture of the GAM and the ARMA time series model. Therefore, it inherits the desirable properties from both models. On one hand, it is as flexible as GAM and is able to describe various relationships between the response variables and the covariates. On the other hand, the proposed GAM explains the autocorrelation structure of the sequential observations. As a result, residuals from (3) can be closer to white noises than those from the conventional GAM, which provide meaningful inputs to the EWMA control chart in Section 4. In terms of estimation methods for (3), the maximum partial likelihood estimation can be used, and the detailed procedure can be found in Benjamin et al.^27^

### 3.2 Dengue case prediction using the proposed GAM model

The proposed GAM is then applied to the phase I dengue count data. Instead of considering the whole dataset in 2006 to 2011, we only consider the weekly counts in 2006, 2008, 2009, and 2010. This is because in these years, the weekly counts are below the corresponding thresholds, as shown in Figure 1. With this treatment, the selected data are approximately in control, which is an essential requirement in the phase I analysis.^12^

To model the count data, a natural way is to assume that *Y*_*t*_ follows the Poisson distribution or the negative binomial distribution, and then to link the conditional mean with the covariates by the GAM.^15^ However, according to our preliminary analysis, neither parametric model fits the dengue count data very well. On the other hand, because the normally distributed residuals are the desirable inputs for the EWMA control chart, a Gaussian error model is developed in this case study. In addition, the weekly index of a year *X*_*t*_=1,…,52 is considered as the covariate to account for the possible seasonal pattern. Moreover, the national population *n*_*t*_ and an AR(2) term are also incorporated into the GAM model. These two terms are commonly considered in dengue prediction models.^28^ In summary, the following GAM is assumed

(4) $$\log(Y_{t})=f_{0}+f_{1}(X_{t})+g_{1}(Y_{t-1})+g_{2}(Y_{t-2})+\log(n_{t})+\epsilon_{t},$$

where *ϵ*_*t*_'s are independent and identically distributed Gaussian errors. In addition, the cubic spline is assumed for all the smooth functions.

**Figure 2:**
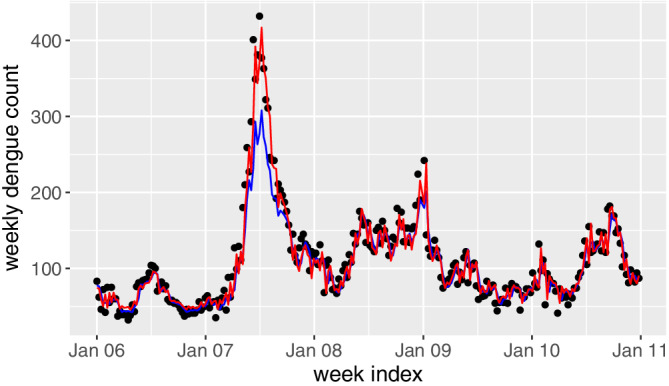
Fitted weekly dengue counts in 2006 to 2011 based on the proposed generalized additive model. The black points denote the observed dengue incidences, the blue line denotes the predicted results by the selected dataset and the red line denotes the predicted results by the whole phase I dataset [Colour figure can be viewed at wileyonlinelibrary.com]

The blue line in Figure 2 shows the fitted one‐week‐ahead dengue counts based on the selected phase I dataset. As a comparison, we also show the predicted counts by using the whole phase I dataset, as shown in red line. Generally, the two lines match well in all the years except for 2007. In 2007, there is a sudden increase in the dengue incidences while the GAM model based on the whole dataset may mask this possible outbreak, as the differences between the observed (black points) and the predicted (red line) values are quite small. If these small differences are put into the control chart, then the outbreak in 2007 cannot be detected. On the contrary, the proposed selective method clearly avoids such a problem. In fact, this example highlights the major difference between the outbreak prediction method and the incidence prediction methods. Generally, most incidence prediction methods in the literature focus on the overall prediction accuracy while the proposed outbreak prediction method aims to detect the rapid and steep increase of dengue incidences.

**Figure 3:**
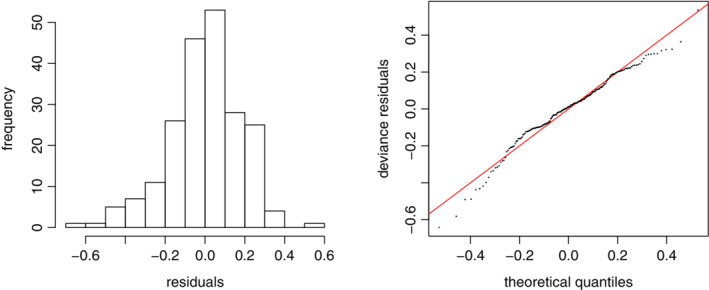
The histogram and the normal quantile‐quantile plots based on the residuals [Colour figure can be viewed at wileyonlinelibrary.com]

**Figure 4:**
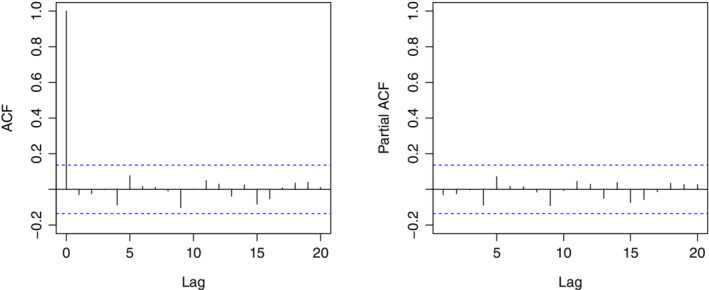
The autocorrelation function (ACF) and partial ACF plots based on the residuals [Colour figure can be viewed at wileyonlinelibrary.com]

We then check the residuals from the proposed model. First, based on the histogram plot and the normal quantile‐quantile plot in Figure 3, the normality assumption of the residuals are verified. Second, we check the independence assumption of the residuals. As seen from the ACF and partial ACF plots of the residuals in Figure 4, there is no significant autocorrelation among the residuals. All these results demonstrate that the proposed GAM can serve as an appropriate model for the dengue count data, and residuals so obtained can be treated as appropriate inputs for a conventional EWMA control chart.

## 4 PHASE II MODELING

Based on the GAM proposed in the last section, the EWMA control chart is used to sequentially monitor the residuals in phase II (eg, 2012‐2017). In specific, for each week during phase II, we predict the (log‐transformed) weekly count by (4) and compute the difference between the predicted and the observed responses. The computed residual is then incorporated into the EWMA control chart to check whether there is a signal of anomaly for the current week. In the following, we first give a brief introduction of the EWMA chart and then show how it could be applied to detect the dengue outbreaks in phase II.

### 4.1 Basics of the EWMA chart

The EWMA control chart consists of plotting a weighted average of measurements (eg, residuals in our example), giving heaviest weights to the most recent observations. This property equips the chart with the ability of detecting moderate sustained shifts in the monitored process. Mathematically, the EWMA statistic *Z*_*t*_ at time *t* is defined as

(5) $$Z_{t}=(1-\lambda )Z_{t-1}+\lambda M_{t},$$

where *M*_*t*_ is the measurement of interest (eg, *ϵ*_*t*_ in our case), and 0<λ≤1 is the smoothing constant. It can be easily shown that (5) could be expressed as

(6) $$Z_{t}=\lambda M_{t}+\lambda (1-\lambda )M_{t-1}+\cdots +\lambda {\left(1-\lambda \right)}^{n-1}M_{1}+{\left(1-\lambda \right)}^{n}M_{0}.$$

As seen, the factor λ controls the weights put on the past observations and the current observation. The smaller the value of λ, the more weights are assigned to the past observations and the less weights are assigned to the current observation. Generally, there are no uniform guidelines for selecting λ, and some commonly used values of λ include 0.05, 0.1, and 0.2.^12^ In cases of detecting upward mean shift (eg, detecting dengue outbreak), the EWMA chart gives signals when *Z*_*t*_ exceeds the control limit *U*, which can be computed by λ and the average run length in control ( ARL_0_). The ARL_0_ means the average run length the chart gives a false signal when the process is in control, and it is determined by the practitioners before implementing the chart.

### 4.2 Dengue outbreak detection using the proposed EWMA chart

**Figure 5:**
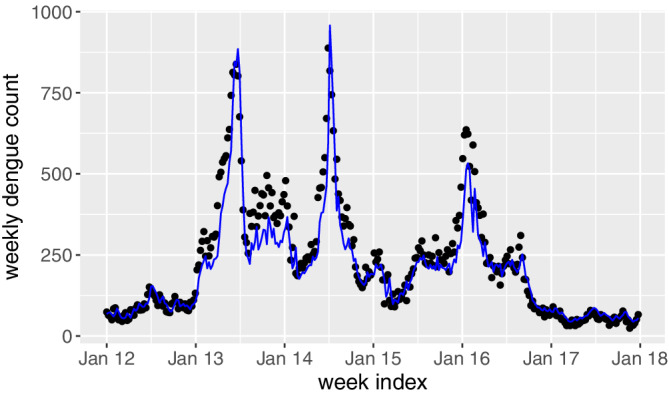
Predicted weekly dengue counts in 2012 to 2017 based on the proposed generalized additive model [Colour figure can be viewed at wileyonlinelibrary.com]

The proposed GAM in Section 3 is first used to make one‐week‐ahead prediction in phase II. The prediction results are shown in Figure 5. As seen, the distance between the predicted and observed counts is relatively large in 2013, indicating possible outbreaks in this period. However, such a visual observation is neither quantitative nor prompt. On the other hand, the EWMA chart is used in this study aiming to provide a quantitative early detection.

The residuals from the GAM model are of interest and hence the EWMA chart is designed as

(7) $$Z_{t}=(1-\lambda )Z_{t-1}+\lambda \epsilon_{t},$$

where *t* is starting from the first week of 2012 and *Z*_0_=0. As discussed in Section 3, the residuals from phase I are approximately normally distributed, that is, *ϵ*_*t*_∼*N*(μ_0_,σ^2^). The values ${\hat{\mu }}_{0}=0$ and $\hat{\sigma }=0.182$ can then be obtained by the maximum likelihood estimation. In phase II, we assume that the distribution of *ϵ*_*t*_ changes to *N*(μ_1_,σ^2^) at some time point *t*, with μ_1_>μ_0_ in presence of an outbreak. Our aim is to detect the mean shift by using the EWMA chart. We standardize the residuals by the estimated ${\hat{\mu }}_{0}$ and $\hat{\sigma }$, and then the control limit *U* for the EWMA chart can be approximately computed as^12^

(8) $$U=\rho \sqrt{\frac{\lambda }{2-\lambda }},$$

where ρ is a parameter depending on λ and *ARL*_0_, and its value can be computed by using the R package “spc.” For some commonly used ARL_0_ and λ, the corresponding values of ρ are shown in Table 2. A signal is given when the monitoring statistic *Z*_*t*_ exceeds *U*.

**Table 2:** The values of *ρ* for some commonly used ARL_0_ and *λ* values

ARL_0_ λ	0.01	0.05	0.1	0.2	0.3	0.4	0.5	0.75
52	0.792	1.431	1.704	1.929	2.027	2.080	2.109	2.123
104	1.109	1.808	2.062	2.255	2.334	2.373	2.394	2.398
156	1.315	2.017	2.254	2.428	2.497	2.531	2.547	2.547
208	1.467	2.159	2.383	2.545	2.608	2.637	2.651	2.648
260	1.586	2.265	2.478	2.631	2.690	2.716	2.728	2.724
312	1.683	2.349	2.554	2.700	2.755	2.780	2.790	2.785
364	1.765	2.417	2.616	2.757	2.809	2.832	2.841	2.835

**Figure 6:**
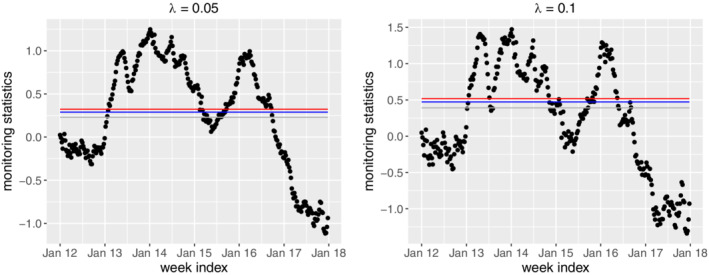
The exponentially weighted moving average control charts under different combinations of ARL_0_ and *λ*. The grey, blue and red lines respectively denote the ARL_0_ values of 52, 104, and 156 [Colour figure can be viewed at wileyonlinelibrary.com]

**Figure 7:**
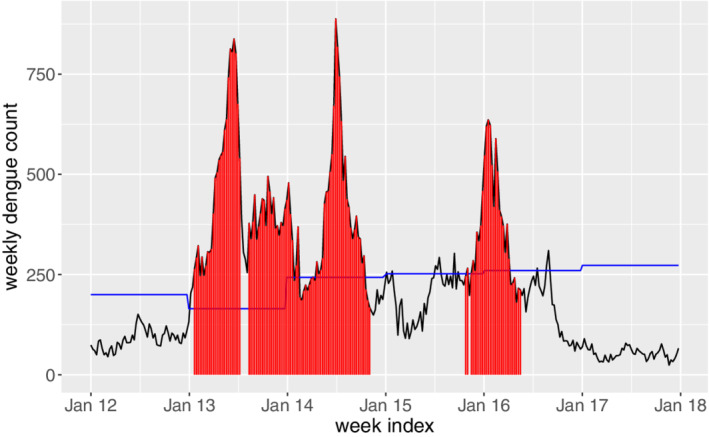
Outbreak signals during 2012 to 2017 given by the exponentially weighted moving average control charts. The blue line denotes the threshold determined by Ministry of Health [Colour figure can be viewed at wileyonlinelibrary.com]

In this case study, we consider ARL_0_=52,104,156, meaning that given the process is in control, there is 1 false alarm per 1, 2, and 3 years, respectively. In addition, we set λ=0.05,0.1. Figure 6 shows the plots of the monitoring statistics *Z*_*t*_ under different combinations of ARL_0_ and λ. As seen, there are clear signals in 2013, 2014 as well as early 2016, which confirm well with the observations from the raw time series data in Figure 1. To see that the EWMA control chart is able to give early warnings, we plot the common signal time points among all the combinations in the original time series, as denoted in red in Figure 7. For comparison purposes, we also show the annul thresholds by MOH, that is, the blue line in Figure 7. As we can see, the EWMA chart gives consistent signals at the early state of the dengue outbreaks in 2013, 2014 and 2016. This is not surprising as the EWMA chart is able to detect small and persistent shift in the process. The persistent‐detection ability is especially helpful in giving warnings of the outbreak in 2014, where a record high of 888 weekly dengue cases was notified. After the outbreak in 2013, the dengue cases seem to decrease in early 2014 and they are below the thresholds, which may mislead the practitioners. However, the proposed EWMA chart keeps generating signals, meaning that another outbreak is probably on the way. As such, taking appropriate interventions may help to prevent the wide spread of dengue from June to August in 2014. In addition, there are weeks in 2015 and 2016 (eg, weeks from June to October in 2015) where the dengue incidences sporadically exceed the thresholds by MOH. If the existing threshold‐based outbreak detection method^24^ is applied, intervention actions should be taken at at those weeks. However, this may be a waste of resource as there is no sudden increase of dengue incidences following those weeks. On the other hand, the proposed framework successfully identifies those weeks as nonoutbreak weeks, which helps in optimizing the control plan. Another interesting finding from Figure 7 is that the EWMA chart may also be used to indicate the end of the outbreak, which is useful in planning and allocating resources.

## 5 SIMULATION STUDIES

We present some simulation results in this section regarding the performance of the proposed GAM and the EWMA chart. Our first simulation considers the comparison with the other commonly used control chart, that is, the cumulative sum (CUSUM) control chart. Based on the standardized residuals *ϵ*_*t*_, the monitoring statistic for a CUSUM chart is

(9) $$C_{t}=\max(0,C_{t-1}+\epsilon_{t}-k),$$

where *k* is a suitable chosen constant. The CUSUM chart gives a signal of upward shift when *C*_*t*_>*U*, where the control limit *U* is determined based on the prefixed *k* and ARL_0_ and it can be computed by using the R package “spc.” Both CUSUM and EWMA charts have been widely used in the context of statistical process control, and the choice between them depends largely on the personnel preference of the user.^12^

We consider generating data from the following GAM model

(10) $$\log(Y_{t})=1.8+\sin(2\pi t/52)+0.2\log(Y_{t-1})+0.3\log(Y_{t-2})+\epsilon_{t},$$

where the sine term considers the seasonal variation and an AR(2) term is incorporated. In phase I, 4‐year observations (ie, a total 4×52=208 weeks) are generated with (*Y*_0_,*Y*_−1_)=(100,90) and *ϵ*_*t*_∼*N*(0,0.1^2^). To ensure the counts being integer, the generated *Y*_*t*_'s are rounded to the smallest integers not less than the original values. In phase II, 3‐year observations are generated. For the first year (ie, *t*=209,…,260), we assume no shift in the mean, that is, *ϵ*_*t*_∼*N*(0,0.1^2^). For the remaining 2 years, a mean shift δ>0 is considered, that is, *ϵ*_*t*_∼*N*(δ,0.1^2^),*t*=261,…,364.

**Figure 8:**
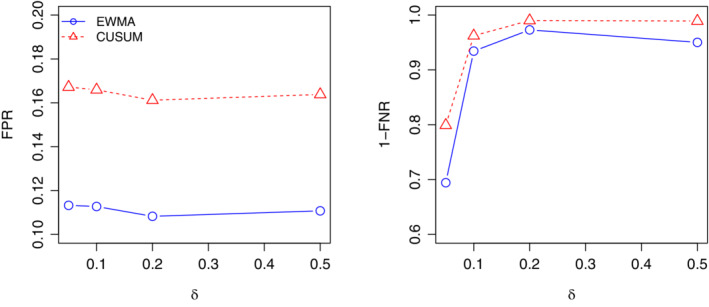
The estimated false positive rate (FPR) and 1‐false negative rate (FNR) under *δ*=0.05,0.1,0.2,0.5 [Colour figure can be viewed at wileyonlinelibrary.com]

The GAM in Section 3 is first developed based on the phase I data, and it is then used to compute the residuals for the phase II data. The residuals are considered as the inputs for the EWMA chart and the CUSUM chart. In this simulation study, we set λ=0.1 in (7) and *k*=0.5 in (9). In addition, a fixed ARL_0_=52 is considered for both charts. We compare the performance of the charts based on the false positive rate (FPR) and the false negative rate (FNR). The FPR is defined as the probability of false signals in an in‐control process, while the FNR is the probability of no signals in an out‐of‐control process. Ideally, smaller values of both FPR and FNR are desirable. However, like the type I error and type II error in a hypothesis test, FPR and FNR are traded each other. In this study, based on 5000 replications, the FPR is estimated based on the first 52 observations in phase II while FNR is estimated based on the remaining 104 observations. Figure 8 shows the estimated values of FPR and 1‐FNR under the mean shift δ=0.05,0.1,0.2,0.5. Generally, the EWMA control chart has a smaller FPR but a larger FNR, compared to the CUSUM chart. In practice, the CUSUM chart may be recommended in detecting very small shift (eg, δ=0.05), as more signals will be generated when the process is out of control. On the other hand, the EWMA chart is appropriate in detecting moderate shift, as it could maintain a lower FPR but a comparable FNR to the CUSUM chart.

Our second simulation aims to verify the robustness of the proposed procedures. In this simulation, we consider generating data from the Poisson regression model, which is a popular model for the count data. In specific, we assume that *Y*_*t*_ follows a Poisson distribution with the mean μ_*t*_, and μ_*t*_ is expressed as

(11) $$\log(\mu_{t})=1.8+\sin(2\pi t/52)+0.2\log(Y_{t-1})+0.3\log(Y_{t-2}).$$

Similar to the first simulation study, 208 observations are generated in phase I with (*Y*_0_,*Y*_−1_)=(100,90). In phase II, the first 52 observations are also from the Poisson distribution with the mean μ_*t*_. For the remaining 104 observations, we consider a mean shift Δ>0 and the count data follows the Poisson distribution with mean μ_*t*_+Δ.

In the literature, the EWMA chart has been used to detect the Poisson mean shift. To obtain the normally distributed inputs, the transformed residuals $e_{t}=(Y_{t}-{\hat{\mu }}_{t})/\sqrt{{\hat{\mu }}_{t}}$ are often used,^19^ and the corresponding EWMA statistics are

(12) $$Z_{t}=(1-\lambda )Z_{t-1}+\lambda e_{t}.$$

**Figure 9:**
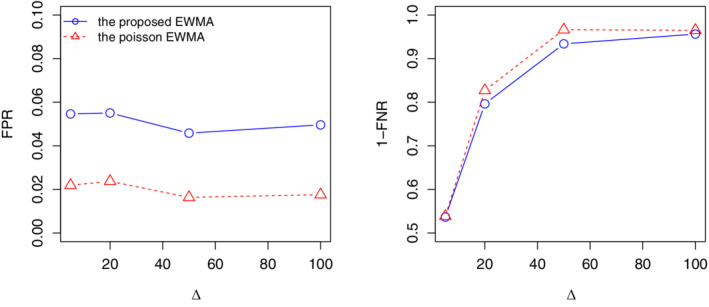
The estimated false positive rate (FPR) and 1‐false negative rate (FNR) under Δ=5,20,50,100 [Colour figure can be viewed at wileyonlinelibrary.com]

Based on the estimation results in phase I, the upper control limit for this EWMA chart can be obtained by the R package “spc.” On the other hand, the EWMA control chart based on the proposed Gaussian error GAM in Section 3 could still be applied to deal with this Poisson distributed dataset. In this simulation, the performance of these two EWMA charts are compared in terms of FPR and FNR. We set λ=0.1 and ARL_0_=52 and consider the Poisson mean shift Δ=5,20,50,100. Figure 9 shows the estimated FPR and 1‐FNR based on 5000 replications. As seen, although the proposed EWMA chart has a slightly higher FPR, its performance in terms of FNR is comparable to the Poisson EWMA chart. This indicates that the proposed detection procedure is quite robust to the underlying data generating mechanism, and it is especially useful when no prior information of the data is available.

**Figure 10:**
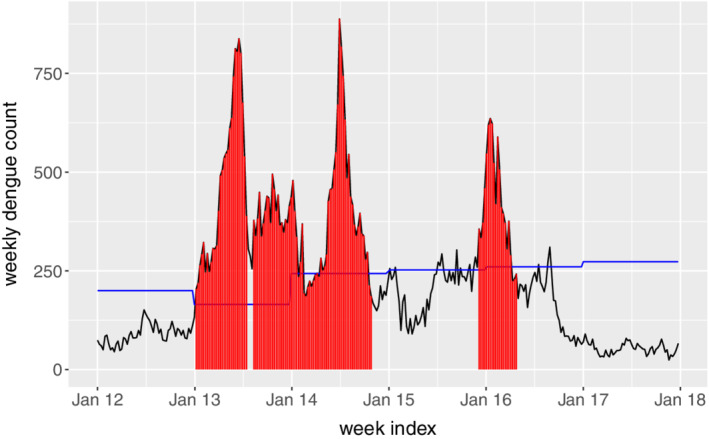
Outbreak signals during 2012 to 2017 given by the cumulative sum control charts. The blue line denotes the threshold determined by Ministry of Health [Colour figure can be viewed at wileyonlinelibrary.com]

**Remark:** We have also applied the CUSUM chart to detect dengue outbreaks by considering the same ARL_0_ values, and the outbreak signals are shown in Figure 10. By comparing with the signals given by the EWMA chart (see Figure 7), we may observe that the two charts perform very similarly in detecting dengue outbreaks, which is consistent with our simulation studies.

## 6 AN ADAPTIVE FRAMEWORK

In the proposed two‐step framework, we selected the “normal" data in phase I by using the existing thresholds, and the weekly data in 2007 and 2011 were discarded. This is reasonable in our case as the peak incidence in 2007 and 2011 are obviously larger than those in the remaining years in phase I. However, since the threshold‐based selection may not be robust in different settings,^24^ it is important to develop a more sophisticated way to determine the normal pattern in phase I. In this section, an adaptive outbreak detection framework which allows automatic selection of the normal pattern in phase I is proposed.

**Figure 11:**
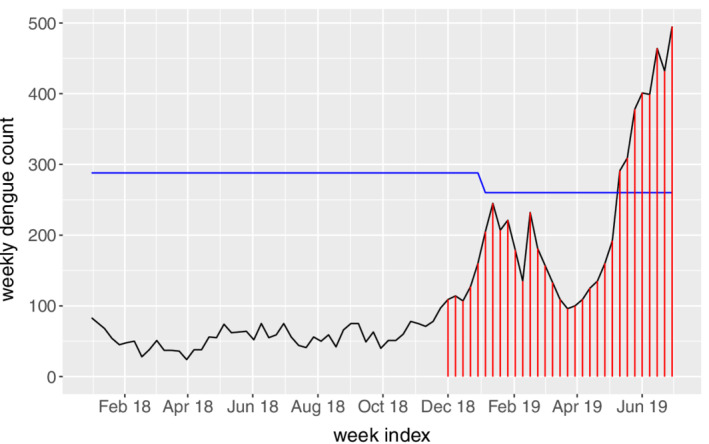
Outbreak signals from January, 2018 to June, 2019 given by the adaptive exponentially weighted moving average control charts. The blue line denotes the threshold determined by Ministry of Health [Colour figure can be viewed at wileyonlinelibrary.com]

The main idea is to use the proposed EWMA chart adaptively. For example, if we want to detect dengue outbreaks from Year 2018 onward, we could first train a new GAM model based on the existing phase II data (ie, 2012‐2017) with the detected outbreak weeks being removed. The new GAM model could then be used in conjunction with the EWMA chart to detect dengue outbreaks for a new phase II period starting from the first week of 2018. This adaptive procedure avoids the use of the threshold and should be effective in different scenarios. To verify the performance, the detected outbreak weeks from January, 2018 to June, 2019 are highlighted in red in Figure 11. As seen, early outbreak signals are given by the proposed adaptive framework before the sudden increase of dengue incidences in 2019, while the thresholds by MOH (blue line) perform quite conservatively.

## 7 CONCLUDING REMARKS

In this study, we developed an early disease outbreak prediction model, and its performance was demonstrated by a case study of predicting dengue outbreaks in Singapore during 2012 to 2017. To our best knowledge, this is the first study focusing on predicting dengue outbreaks by using the statistical control charts. Compared to the weekly dengue incidences prediction models which are usually measured by the average prediction errors over a period, a prediction of dengue outbreaks which correspond to a rapid and steep increase of dengue incidences and cause a surge of cumulative dengue cases over a short period is pivotal for dengue control practitioners. In 2013, the peak weekly dengue incidence in Singapore reached to 838, and there were 5924 cases incurred within 2 months. This apparently challenged the public health systems, especially when there was chikungunya disease also reaching its high incidences during the same period. This calls for an intelligent tool for predicting dengue outbreaks and bringing attentions to agencies and the public for confronting to the subsequent challenge.

In the proposed two‐step prediction model, a GAM was first developed based on the phase I (2006‐2011) data. By incorporating the ARMA terms, the obtained residuals were shown to be independent and identically distributed normal, which enables the use of the conventional EWMA control chart in phase II (2012‐2017). The proposed EWMA chart is able to capture the historical dengue incidence patterns and distinguish between endemic situations and the outbreak patterns. It could automatically generate outbreak signals, which are often more reasonable than the threshold‐based outbreak signals. Through the case study, we show that the proposed framework is very effective in detecting abnormal dengue patterns based on the temporal incidence data and it successfully gave early warnings of the severe dengue outbreaks in 2013, 2014, and 2016. Such prediction capability provides opportunity for public health officers to take prompt interventions before the wide spread of the disease.

For further demonstrating performance of the proposed outbreak prediction model, we carried out simulation studies, in which we compared the proposed EWMA chart with the other commonly used control chart, for example, the CUSUM chart. The simulation results showed that our proposed EWMA chart works better than the CUSUM chart in detecting moderate and large shifts in the monitored process, which is essential in dengue outbreak detection. In addition, the proposed two‐step detection procedure considers the training and learning process based on the historical dengue incidences and the prediction process for outbreak signal detection and prediction. Hence, it could be easily used regardless of the underlying distribution of the data.

In practice, it is of interest to investigate when intervention should be taken given the first appearance of an outbreak signal. Mathematically, an outbreak signal means the monitoring statistics exceed the control limit and hence the appropriate interventions should be taken immediately. In practical use, however, a single outbreak signal may be a false alarm and taking interventions may be quite costly. In view of this fact, we suggest taking interventions in presence of three consecutive signals. This is because if we set 1 false alarm per year in the model, the probability of nonoutbreak given three consecutive signals would approximately be 7.1×10^−6^, which is negligible in practice.

Because the proposed two‐step framework is quite general, it should also work well for detecting outbreaks of other diseases such as salmonellosis, malaria, and Influenza. In such cases, it is possible to incorporate other covariates such as weather factors to achieve a more accurate estimation. In addition, it is of interest to detect disease outbreak in a specific location. We believe substantial efforts are needed to first investigate the spatiotemporal patterns of the disease and then to detect the possible outbreaks, which is one of our future research focuses. Another possible future research direction is about how to determine the two phases in practice. In this study, we demonstrate the proposed framework by separating the data into two phases. We consider the period 2012 to 2017 in phase II as these years have witnessed serious dengue outbreaks, and hence the usefulness of the proposed framework can be better illustrated. However, it is important to develop some strategies to determine the two phases in practice. Generally, a long time period of phase I is required to ensure that an adequate GAM model can be trained. In addition, the GAM model should be updated regularly so that it can capture the most recent pattern of the disease propagation. In other words, a single and fixed GAM model should not be applied for a long time period and hence the duration of phase II should be short. Based on the above discussion, we suggest that the duration of phase I should not be less than 5 years and the duration of phase II may be set as 1 year in practice. That is, at beginning of each year, we may first use the data in the last five years to train the GAM model and then detect outbreaks by the EWMA chart sequentially as the weekly data in the new year become available. With this treatment, the GAM model is updated at the beginning of each year and it serves as the one‐week‐ahead prediction model in the coming year. We believe this ad hoc strategy is helpful in applying the proposed framework in practice. Nevertheless, more rigorous treatment of this issue is worth further investigation.
